# Newly Isolated Bacteriophages from the *Podoviridae*, *Siphoviridae*, and *Myoviridae* Families Have Variable Effects on Putative Novel *Dickeya* spp.

**DOI:** 10.3389/fmicb.2017.01870

**Published:** 2017-09-28

**Authors:** Špela Alič, Tina Naglič, Magda Tušek-Žnidarič, Maja Ravnikar, Nejc Rački, Matjaž Peterka, Tanja Dreo

**Affiliations:** ^1^Department of Biotechnology and Systems Biology, National Institute of Biology, Ljubljana, Slovenia; ^2^Jožef Stefan International Postgraduate School, Ljubljana, Slovenia; ^3^Laboratory for Bioanalytics (LBA), Centre of Excellence for Biosensors, Instrumentation and Process Control (COBIK), Ajdovščina, Slovenia

**Keywords:** *Dickeya*, bacteriophages, *Podoviridae*, genome sequencing, resistance development, convective interaction media monolith chromatography

## Abstract

Soft rot pathogenic bacteria from the genus *Dickeya* cause severe economic losses in orchid nurseries worldwide, and there is no effective control currently available. In the last decade, the genus *Dickeya* has undergone multiple changes as multiple new taxa have been described, and just recently a new putative *Dickeya* species was reported. This study reports the isolation of three bacteriophages active against putative novel *Dickeya* spp. isolates from commercially produced infected orchids that show variable host-range profiles. Bacteriophages were isolated through enrichment from *Dickeya*-infected orchid tissue. Convective interaction media monolith chromatography was used to isolate bacteriophages from wastewaters, demonstrating its suitability for the isolation of infective bacteriophages from natural sources. Based on bacteriophage morphology, all isolated bacteriophages were classified as being in the order *Caudovirales*, belonging to three different families, *Podoviridae*, *Myoviridae*, and *Siphoviridae*. The presence of three different groups of bacteriophages was confirmed by analyzing the bacteriophage specificity of bacterial hosts, restriction fragment length polymorphism and plaque morphology. Bacteriophage BF25/12, the first reported *Podoviridae* bacteriophage effective against *Dickeya* spp., was selected for further characterization. Its genome sequence determined by next-generation sequencing showed limited similarity to other characterized *Podoviridae* bacteriophages. Interactions among the bacteriophages and *Dickeya* spp. were examined using transmission electron microscopy, which revealed degradation of electron-dense granules in response to bacteriophage infection in some *Dickeya* strains. The temperature stability of the chosen *Podoviridae* bacteriophage monitored over 1 year showed a substantial decrease in the survival of bacteriophages stored at -20°C over longer periods. It showed susceptibility to low pH and UV radiation but was stable in neutral and alkaline pH. Furthermore, the stability of the tested bacteriophage was also connected to the incubation medium and bacteriophage concentration at certain pH values. Finally, the emergence of bacteriophage-resistant bacterial colonies is highly connected to the concentration of bacteriophages in the bacterial environment. This is the first report on bacteriophages against *Dickeya* from the *Podoviridae* family to expand on potential bacteriophages to include in bacteriophage cocktails as biocontrol agents. Some of these bacteriophage isolates also showed activity against *Dickeya solani*, an aggressive strain that causes the soft rot of potatoes, which indicates their broad potential as biocontrol agents.

## Introduction

*Dickeya* spp. (formerly *Erwinia chrysanthemi*) are plant pathogenic bacteria that can cause soft rot disease across a wide range of crops and ornamental plants worldwide (Czajkowski et al., 2011; Toth et al., 2011; Adriaenssens et al., 2012). These Gram-negative, facultative anaerobic bacteria of the γ-*Proteobacteria* subdivision cluster into the *Enterobacteriaceae* family. *Dickeya* characteristically produce cell wall-degrading enzymes that are secreted during their infection of plants, resulting in the plant–tissue maceration that is typical of soft rot disease (Czajkowski et al., 2011; Adriaenssens et al., 2012). *Dickeya* can be spread over long distances via infected plants and can also live as epiphytes or facultative saprophytes in soil and ground water (Reverchon and Nasser, 2013). They have been detected in a variety of water sources, and irrigation water has been reported as the probable source of their infection of potatoes in Australia (Toth et al., 2011). There are currently no effective chemical agents to control *Dickeya* soft rot infection, resulting in significant economic loss, particularly in terms of potato production (Adriaenssens et al., 2012; Czajkowski et al., 2014).

The genus *Dickeya* currently encompasses eight species—*Dickeya zeae*, *D. dadantii*, *D. chrysanthemi*, *D. solani*, *D. aquatica*, *D. dianthicola*, *D. paradisiaca*, and *D. fangzhongdai* (Samson et al., 2005; Toth et al., 2011; Adriaenssens et al., 2012; Tian et al., 2016). The use of molecular tools has revealed further evolution of these species, with the occasional description of strains with characteristics different from the previously described species, such as unassigned lineages (UDLs), in the *Dickeya* phylogenetic backbone (Samson et al., 2005; Parkinson et al., 2009; Adriaenssens et al., 2012; Van Vaerenbergh et al., 2012). Van Vaerenbergh et al. (2012) reported the introduction of UDLs, mostly for *Dickeya* isolates from ornamental plants. Furthermore, some of the isolates corresponding to UDL-3 and UDL-4 based on the *fliC* sequence were described as putative new species in the genus *Dickeya* (Alič et al., 2017).

The use of bacteriophages as biocontrol agents is an attractive option for controlling bacterial diseases of plants (Jones et al., 2007; Adriaenssens et al., 2012; Buttimer et al., 2017). Bacteriophages can show high specificity for the bacterial hosts they infect, and only lyse bacterial cells, thus providing targeted disease management. Bacteriophages are naturally present in the environment, such as in soil, water, plants, and animals, where they can persist through host-dependent self-replication over long periods. As bacteriophages cannot infect eukaryotic cells, they are safe to use (reviewed in Jones et al., 2007). While only lytic bacteriophages can be used for bacteriophage therapy and biocontrol (Jones et al., 2007, 2012), their relatively easy and inexpensive production means that they are of commercial interest (reviewed in Jones et al., 2007).

Despite these advantages, few bacteriophages have demonstrated repeated, successful applications in plant disease management. Apart from the obvious challenges, such as resistance development in bacteria (Katznelson, 1937; Levin and Bull, 2004), bacteriophage therapies must take into account the complex dynamics among bacteriophages, bacteria and their environment, which largely remain unexplored (Levin and Bull, 2004). In addition, suitable protective formulation and delivery of bacteriophages to affected plants must be optimized to ensure their efficient application and their survival during this application (Jones et al., 2007; Frampton et al., 2012).

The possibility of plant disease control using bacteriophages has been studied for various plant pathogenic bacteria (e.g., *E. amylovora*, *Agrobacterium tumefaciens*, *Ralstonia solanacearum*, *Streptomyces scabies, Pseudomonas* sp.*, Xanthomonas* sp., *Pectobacterium* sp., *Xylella fastidiosa*, and *Dickeya* sp.) (reviewed in Buttimer et al., 2017). The idea of using bacteriophages as controlling agents for *Dickeya* spp. has been proposed before, but only limited attempts have been made to isolate such lytic bacteriophages (Czajkowski et al., 2014). Adriaenssens et al. (2012) described the T4-related bacteriophages LIMEstone1 and LIMEstone2 that specifically infect *D. solani*. In their study, both bacteria and bacteriophages were isolated from soil taken from a potato field. The first reported experimental field trial for the bacteriophage treatment of potato tubers infected with *D. solani* resulted in improved yields, which led to the proposal of the bacteriophages LIMEstone1 and LIMEstone2 as effective therapeutics in an agricultural setting (Adriaenssens et al., 2012). Czajkowski et al. (2014) isolated and identified nine more bacteriophages that showed wider host ranges and that were all active against *D. solani*. All these bacteriophages prevented the growth of *D. solani in vitro* and protected potato tuber tissue from the maceration caused by *D. solani*.

However, further studies are required to determine the long-term effectiveness of bacteriophages and to perform field trials. Some bacteriophages with broader host ranges have been reported to infect some *Pectobacterium* strains as well as *D. solani* (Czajkowski et al., 2015). Recently, bacteriophages that are active against *D. dadantii* isolates were isolated from potato plants. These bacteriophage isolates are members of the *Myoviridae* and *Siphoviridae* families and were all isolated from the water of the Caspian Sea. Their effectiveness was tested *in vitro*, and they showed promising biocontrol potential. However, no field trials have been conducted to date (Soleimani-Delfan et al., 2015).

The variously reported investigations and trials involving *Dickeya* spp. have so far only been carried out in terms of improving potato production (Adriaenssens et al., 2012; Czajkowski et al., 2014). However, ornamental plants with high added value, such as orchids, have significant economic importance in agricultural production, and due to extensive worldwide trade, if infected, these can facilitate the spread and possible expansion of the pathogenic bacteria host range. The aim of the study was to isolate and characterize bacteriophages active against putative new *Dickeya* spp. as a research tool to study the bacteria–bacteriophage system and as a potential biocontrol component.

Here, we report on the successful application of the convective interaction media (CIM) method for bacteriophage isolation from complex environmental samples. We show the activity of bacteriophage isolates against two previously determined putative *Dickeya* spp. (UDL-3 and UDL-4; Alič et al., 2017). For the isolates, we determine their host range and restriction fragment polymorphism (RFLP) profiles and characterize them according to their morphology using transmission electron microscopy. Finally, we describe the first reported *Podoviridae* bacteriophage effective against *Dickeya* spp., BF25/12, including its annotated genome sequence.

## Materials and Methods

### Bacterial Strains and Media

*Dickeya* strains were isolated from *Phalaenopsis* orchid leaves that showed soft rot symptoms and were identified as a putative new species within the genus *Dickeya* (Alič et al., 2017). Based on the *fliC* gene sequence, the bacteria isolates were classified as members of UDL-3 and UDL-4 (Alič et al., 2017). These bacteria were routinely grown on Luria Bertani (LB) medium with 1.5% agar and 0.05% (w/v) NaCl at 28–30°C. For liquid preparations, the bacteria cultures were grown in LB broth at 28°C, with agitation at 200 rpm unless otherwise stated. LB with 0.4% agar was used for overlays. For long-term storage, the bacteria cultures and isolates were kept in a bacterial and fungal storage system (Microbank; bioTRADING, Mijdrecht, Netherlands) at ≤-76°C.

### Isolation of Bacteriophages

#### Isolation of Bacteriophages from Plant Material

Bacteriophages against *Dickeya* UDL-3 were isolated from *Phalaenopsis* orchid leaves that showed soft rot symptoms. The orchids were obtained from a commercial orchid production site. Extracts of the plant tissues were prepared by commuting or macerating plant material in 45 mL of Trypticase Soy Broth (30 g Trypticase Soy Broth [BD 211768], double-distilled water to 1 L, pH 9). Sterile 450 μL 1 M MgSO_4_ and 90 μL 1 M CaCl_2_ were added to the mixture. The extract was incubated for 1 h at room temperature with constant rotation (Heto Mastermix rotator), followed by centrifugation at 10,000 × *g* for 10 min. The resulting supernatant was carefully poured into new 50 mL tubes (Falcon). The clear extract was again centrifuged as above. Finally, the supernatant was sterile filtered through 0.2-μm membranes (Millipore) and stored at 4°C until enrichment. Enrichment of the bacteriophages in the extract was performed with a mixture of different *Dickeya* isolates (**Table 1**). For enrichment, 15 mL double-strength LB (20 g tryptone, 10 g yeast extract, 1 g NaCl, double-distilled water to 1 L) with 300 μL 1 M MgSO_4_ and 60 μL 1 M CaCl_2_ was mixed with 15 mL extract of bacteriophages. To this, 150 μL bacteria in log phase growth (12–14 h incubation at 28°C, with shaking at 200 rpm) was added at an equal ratio (Van Twest and Kropinski, 2009). This was incubated for approximately 24 h at 28°C, with shaking at 50 rpm.

**Table 1:** *Dickeya* spp. isolates used in the bacteriophage enrichment.

Sample	Bacteria mixture	*Dickeya* spp. isolates used
Diseased orchid	UDL-3	B16, COB2/12, COB7/12, COB8/12, COB10/12, COB11/12, COB11/12, COB15/12, COB16/12 COB17/12
Tissue	UDL-4	S1, COB9/12, COB12/12, COB14/12
Wastewater	UDL-3	COB10/12, COB11/12, COB16/12
	UDL-4	COB9/12, COB12/12, COB14/12

*For plant material bacteriophage enrichment, only one mixture was used, which contained all the bacteria isolates. The wastewater bacteriophage enrichment was conducted separately for the UDL-3 and UDL-4 bacteria mixtures*.

The enriched suspension (10 μL) was spotted on a streak of bacteria in log phase growth used in the enrichment and on a plate with an LB overlay that contained the same bacteria isolates, following Van Twest and Kropinski (2009). After a 24-h incubation at 28°C, the resulting lysis cones were scratched into 200 μL saline-magnesium with gelatine (SMG; 5.8 g NaCl, 2.0 g MgSO_4_ × 7H_2_O, 7.88 g Tris HCl, pH 7.5, 0.1 g gelatine, double-distilled water to 1 L), and double agar overlay plaque assays were performed (Kropinski et al., 2009). The host bacterium for the plaque assays was prepared as follows: 1 μL inoculation loop of overnight bacteria grown on solid LB was transferred to 5 mL liquid LB with 50 μL 1 M MgSO_4_ and 10 μL 1 M CaCl_2_ and incubated for 12–14 h at 28°C, with shaking at 100 rpm. The overnight culture was then diluted 10-fold to obtain the optical density at 600 nm (OD_600_) of 0.5, which corresponded to approximately 10^8^ cells per mL. Single plaques were selected and transferred to SMG for further purification using disposable pipette tips. The bacteriophages were purified by streaking 1 μL bacteriophage suspension on solid LB and pouring over the indicator bacterial lawn. Three successive single plaque isolations were performed as described above. For bacteriophage amplification, double agar overlay plaque assays on LB were carried out (enumeration of bacteriophages by double agar overlay plaque assay), following Kropinski et al. (2009) but with several modifications: (i) 2 mL of the top agar was dispensed into sterile tubes; (ii) tubes containing top agar were incubated at 52°C and (iii) 63 μL bacteria suspension and bacteriophage dilution were mixed and incubated for 20 min at room temperature prior to the transfer to the warm overlay medium, with agitation at 150 rpm. Confluent plates (plaques evenly distributed over the plates as single plaques and not touching others) were then poured with 3 mL SMG. The plates were sealed with Parafilm and incubated for approximately 20 h at +4°C, with gentle shaking at 100 rpm. The bacteriophages were sterile filtered over 0.2-μm membranes and stored at +4°C or at -80°C for longer periods.

#### Isolation of Bacteriophages from Wastewater

Bacteriophages against the *Dickeya* UDL-3 and UDL-4 strains were isolated from wastewater concentrates. Effluent water was collected from a wastewater treatment plant and concentrated using a CIM quaternary amine monolithic column. The preparation and characterization of the water samples were as described in Steyer et al. (2015). The wastewater bacteriophage concentrates were diluted in double-distilled water (1:2; v/v) to a final 1% NaCl.

For the enrichment, 3 mL double-strength LB with 120 μL 1 M MgSO_4_ and 24 μL 1 M CaCl_2_ was mixed with 3 mL diluted bacteriophage concentrate. To that, 30 μL of bacteria in log phase (12–14 h incubation at 28°C, with shaking at 200 rpm) was added at an equal ratio (Van Twest and Kropinski, 2009). The enrichment of UDL-3 and UDL-4 bacteria (**Table 1**) was preformed separately. The enrichments were incubated for approximately 24 h at 28°C, with shaking at 50 rpm and sterile filtering through 0.2-μm membranes.

The bacteriophage suspension (7 μL) was used in the enrichment on plates with an LB overlay, with plaques isolated as described for the plant material isolation. The bacteriophages were purified by repeating the plaque assays three times. For bacteriophage amplification, double agar overlay plaque assays were carried out as described above. The bacteriophages were eluted from confluent plates with the change of the incubation temperature to room temperature (25°C) and were stored as described above.

### Host Range and Specificity of Bacteriophages

For host-range determination, 56 bacterial strains and isolates (**Table 4**) were tested. This included 14 *Dickeya* isolates from diseased *Phalaenopsis*, eight *Dickeya* spp. reference strains from a culture collection (*D. solani*, *D. dieffenbachiae*, *D. dianthicola*, *D. zeae*, *D. dadanti*, *D. aquatica*, two *Dickeya* spp.), seven *Pectobacterium cypripedii* from a culture collection, four non-*Dickeya* spp. isolates from diseased *Phalaenopsis*, 10 isolates from healthy *Phalaenopsis* leaves and 14 different bacterial strains from the *Enterobacteriaceae* family. The host range was tested by spotting 10 μL of a bacteriophage suspension onto an LB bacterial lawn, which was performed as three biological repeats. All positive results were confirmed by double agar overlay plaque assays (described above) to eliminate false positives.

### Transmission Electron Microscopy

The presence and morphology of the isolated bacteriophages were determined using transmission electron microscopy with a negative staining method. Here, 20-μL bacteriophage suspensions were deposited on Formvar-coated and carbon-stabilized copper grids and stained with a 1% (w/v) aqueous solution of uranyl acetate.

To accompany the bacteria–bacteriophage interactions, bacterial suspensions (i.e., UDL-3 and UDL-4 separately) were mixed with the purified selected bacteriophage BF25/12, which was shown to be active against bacteria and was kept for 15 min at room temperature. The suspensions were centrifuged at 3,000 × *g* for 10 min. The supernatants were carefully removed, and the pellets were resuspended in 3% glutaraldehyde and 1% paraformaldehyde in 0.1 M Sörensen phosphate buffer (Merck), pH 7.2, for 20 h at 4°C. For UDL-3, pieces of soft agar that contained borders of plaques from the plaque assay and soft agar with bacteria from the control plate were fixed in the same fixative. All the samples were post-fixed in 2% OsO_4_ in 0.1 M Sörensen phosphate buffer (Merck) pH 7.2 and embedded in Agar 100 resin (Agar Scientific). Ultrathin sections were stained with 2% (w/v) aqueous uranyl acetate and Reynolds lead citrate (Agar Scientific).

The grids were observed using transmission electron microscopy (CM 100; Philips, Netherlands) operated at 80 kV and equipped with a CCD camera (Orius SC 200) and Digital Micrograph software (Gatan Inc., United States).

### Bacteriophage Genome Characterization

Bacteriophage DNA was isolated according to Pickard (2009) using the Phase Lock Gel system (5 PRIME, Germany). To determine the diversity of the isolated bacteriophages, RFLP analysis was performed according to Adriaenssens et al. (2012) using the Hind II (Thermo Scientific) restriction enzyme. Here, 0.5 μg bacteriophage DNA was used per reaction mixture. The restriction was performed at 37°C for 16 h. The fragments were analyzed on 0.8% agarose gels for 100–120 min at 90 V. Bacteriophage BF25/12 was selected from among *Podoviridae* isolates for further characterization.

### Whole Genome Sequencing

The whole genome of bacteriophage BF25/12 was sequenced using 454 sequencing technology. The paired-end library was sequenced at Microsynth (Balgach, Switzerland) following the manufacturer’s instructions to 36-fold coverage. Whole genome assembly was performed with the GS De Novo Assembler using default parameters (Microsynth). The fully assembled genome was annotated using Rapid Annotations using Subsystems Technology (RAST) (Aziz et al., 2008; Overbeek et al., 2014; Brettin et al., 2015) and the default setting options. Additionally, genome annotation was verified and curated by BLAST analysis (Altschul et al., 1990). The assembled genome sequence was compared to other characterized bacteriophages using the PASC web tool (Bao et al., 2014) and BLAST analysis (Altschul et al., 1990).

### UV Radiation Stability

Bacteriophage stability was tested for bacteriophage BF25/12. A bacteriophage suspension was prepared in SMG buffer, and the bacteriophage concentration was adjusted to the concentration of 10^8^ pfu/mL. Testing was performed on 5 mL of prepared bacteriophage suspension in open Petri dishes (Φ = 55 mm, Golas) illuminated using double UV-C light (Philips TUV G30T8, UV dose approximately 100 mJ/cm^2^ 30 cm from the light source) for 1 and 2 min. The Petri dishes were kept on ice covered with cloth to minimize the influence of heat produced by the UV light. The bacteriophage stability was tested by double agar overlay plaque assay (described above) in two biological repeats as two technical replicates.

### pH Stability

The pH stability of the bacteriophage BF25/12 was tested for a selected range of pH values from 3 to 11. Testing was performed in SMG buffer and sterile demineralised water, adjusted with HCl or NaOH to the pH values of 3, 5, 7, 9, and 11. Phage suspensions were mixed with buffer or water (1 ml total volume) for final concentrations of 105 and 103 pfu/mL and were incubated at room temperature with shaking (100 rpm) for 24 h. The concentration of active bacteriophages was determined with a double agar overlay plaque assay (described above) in two biological repeats and two technical replicates.

### Temperature Stability

Bacteriophage stability was analyzed for the bacteriophage BF25/12 by incubating a bacteriophage suspension (100 μL) in SMG at different temperatures, +4, +28, -20, and -80°C. The stability was tested once per month for 1 year using double agar overlay plaque assays (described above) as three technical replicates.

### Development of Bacterial Resistance

The emergence of bacterial resistance was measured for the combination of the selected bacteriophage BF25/12 and the bacteria *Dickeya* sp. B16. A double agar overlay assay was used to enumerate the emergence of resistant bacterial colonies at a bacterial concentration of 10^8^ cfu/mL and four different bacteriophage concentrations, 10^3^, 10^4^, 10^5^, and 10^6^ pfu/mL. Resistant bacterial colonies were counted on plates, with double agar overlay plaque assays carried out after 27 h of incubation at 28°C. The test was performed in triplicate per bacteriophage concentration, with two technical repeats each.

## Results

### Sourcing of Bacteriophages and Characterization

The target *Dickeya* spp. bacteria used in this study were previously isolated from diseased *Phalaenopsis* orchids and identified as two distinct UDLs, UDL-3 and UDL-4, based on the partial sequencing of the *fliC* and *dnaX* genes (Alič et al., 2017). Together with a set of reference bacteria (**Table 1**), these were used as a mixture to enrich bacteriophages from extracts of rotting orchid leaves and wastewater. Altogether, 18 bacteriophages against *Dickeya* spp. were isolated, five from diseased *Phalaenopsis* leaves and 13 from wastewater. Bacteriophages active against UDL-3 bacteria were successfully isolated from plant tissue, but bacteriophages with strong activity against UDL-4 were only isolated from municipal wastewater far away from the orchid site of production. Bacteriophages were isolated from wastewater using a concentration of the bacteriophage particles with CIM monolith chromatography. Together with the subsequent plaque assays, this demonstrates the suitability of this approach for the isolation of infective bacteriophages from wastewater samples.

**FIGURE 1:**
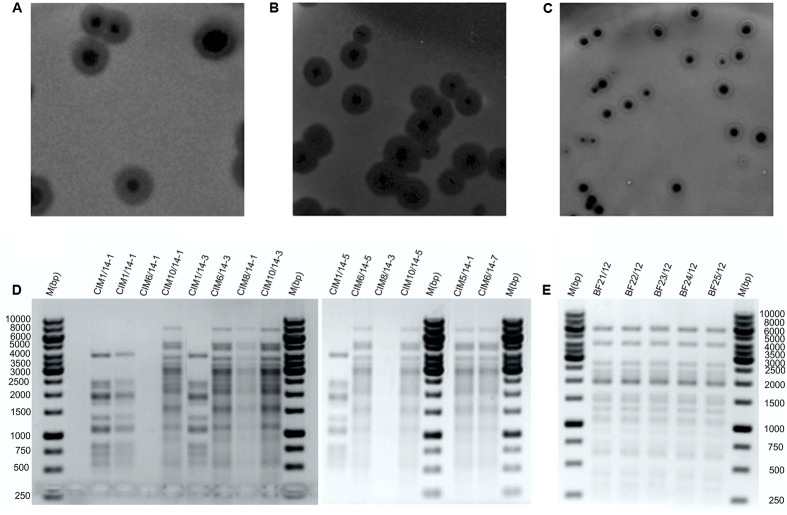
Representative bacteriophage plaque morphologies and restriction fragment length polymorphism profiles. Plaque morphologies for bacteriophages isolated from plant material **(A)** and wastewater **(B,C)**. Bacteriophages against *Dickeya* spp. isolated from wastewater **(D)** and from diseased *Phalaenopsis* **(E)** digested with Hind II restriction enzyme. M, Gene Rule 1 kb DNA ladder.

The bacteriophage recovery from municipal wastewater varied during the season of monthly sampling and the isolation attempts. Bacteriophages active against *Dickeya* spp. were predominantly isolated in the warmer months (**Table 2**). After overnight incubation, all the bacteriophages active against *Dickeya* spp. from diseased *Phalaenopsis* isolates produced large clear plaques of approximately 6 mm in diameter with a halo effect, suggesting the presence of lysins (**Figure 1A**). Mixed bacteriophage populations were ruled out by purifying the bacteriophages separately from the clear plaque zones and the halo rings, which resulted in identical morphology with a halo effect for all of them. Furthermore, the restriction of bacteriophage DNA with Hind II (**Figure 1E**) showed no differences in the patterns between the bacteriophages isolated from these diseased *Phalaenopsis* orchids.

**Table 2:** Sampling timeline of the plant material and wastewater.

Month	Mean monthly temperature^a^ (°C)	Diseased orchid tissue		Wastewater	
		Sampled	Bacteriophage isolated	Sampled	Bacteriophage isolated
January	1.6	-	-	+	-
February	-0.8	-	-	+	-
March	10.1	+	-	+	-
April	11.4	+	-	+	+
May	16.1	+	-	+	+
June	21.3	-	-	+	+
July	22.7	+	+	+	-
August	23.3	-	-	+	+
September	17.0	+	+	+	-
October	11.7	-	-	+	+
November	8.8	-	-	+	-
December	0.8	+	-	+	-

^a^*Data derived from the Republic of Slovenia Statistical Office RS SI-Stat Data Portal (http://pxweb.stat.si/pxweb/dialog/statfile1.asp)*.

The bacteriophages active against *Dickeya* spp. that were isolated from wastewater showed two different plaque morphologies. The bacteriophages from the June samples produced large clear plaques that were approximately 5 mm in diameter with a halo effect, and those isolated in April, May, August, and October produced medium to small plaques of approximately 3 mm in diameter, with a clear zone encircled by a white ring and a halo effect (**Figures 1B,C**). Restriction of the bacteriophage DNA derived from the wastewater showed two different RFLP patterns (**Figure 1D**). The bacteriophages BF-CIM1/14 that were isolated in June had a uniform RFLP pattern that differed from all the other bacteriophages isolated from wastewater and instead showed a common RFLP pattern.

**FIGURE 2:**
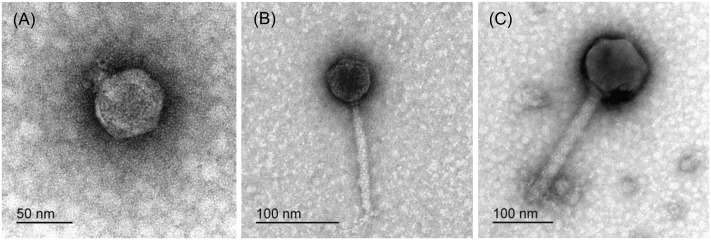
Representative transmission electron microscopy micrographs of bacteriophages isolated from diseased *Phalaenopsis* leaves **(A)** and sewage water **(B,C)**, all of which were only active against *Dickeya* spp.

Based on the morphologies of the bacteriophage particles examined with transmission electron microscopy (using negative staining), two of five isolated bacteriophages from diseased *Phalaenopsis* were placed in the *Podoviridae* family and showed a head size of 55.8 ± 2.3 nm × 52.6 ± 3.1 nm (*n* = 20) (**Figure 2A**). The bacteriophage tail dimensions were difficult to determine accurately because whole tails were rarely seen and the size was highly dependent on the bacteriophage position on the grid. However, the tail size was estimated to be around 12.1 ± 2.4 nm × 16.3 ± 1.7 nm (*n* = 13) (**Figure 2A**). The bacteriophages from wastewater were classified as *Siphoviridae* (**Figure 2B**) and *Myoviridae* (**Figure 2C**), which are both in the order Caudovirales. Bacteriophages from the *Siphoviridae* family (**Figure 2B**) had icosahedral heads (54.6 ± 2.6 nm × 53.9 ± 2.2 nm, *n* = 24) and slightly flexible tails (131.7 ± 8.4 nm × 10.6 ± 0.6 nm, *n* = 23) that continued into thin terminal fibers. Bacteriophages characterized as members of the *Myoviridae* family (**Figure 2C**) had larger icosahedral heads (138.2 ± 3.7 nm × 136.6 ± 4.1 nm, *n* = 47) and rigid contractile tails (228.1 ± 7.0 nm × 24.8 ± 1.4 nm, *n* = 47). The base plates were rather large (approximate length of 30 nm), and the attached spikes were short and could be observed only criss-crossed in a netlike structure and were therefore impossible to count. In summary, all these bacteriophages active against *Dickeya* spp. belong to one of three groups, having common RFLP profiles and plaque and bacteriophage morphologies (**Table 3**).

**Table 3:** Bacteriophages isolated from infected *Phalaenopsis* tissue and wastewater concentrates using convective interaction media (CIM) monolith chromatography.

Sample origin	Bacteriophage code	Plaque description	Bacteriophage	family
*Phalaenopsis*	BF 21/12	Large clear	*Podoviridae*
Tissue	BF 22/12	plaques with	
	BF 23/12	halo effect	
	BF 24/12		
	BF 25/12		
Wastewater	CIM 1/14-1	Large clear	*Siphoviridae*
	CIM 1/14-3	plaques with	
	CIM 1/14-5	halo effect	
	CIM 5/14-1	Medium to small	*Myoviridae*
	CIM 6/14-1	plaques with clear	
	CIM 6/14-3	cone encircled	
	CIM 6/14-5	by white ring	
	CIM 6/14-7	and halo effect	
	CIM 8/14-1		
	CIM 8/14-3		
	CIM 10/14-1		
	CIM 10/14-3		
	CIM 10/14-5		

### Specificity of Isolated Bacteriophages against *Dickeya* spp.

The specificities of the interactions between the bacteria and bacteriophages were examined using spot tests (i.e., isolation of bacteriophage via induction of lysogenesis) and were confirmed using plaque assays. The specificities of the bacteriophages included in the testing were limited to *Dickeya* spp., excluding *D. aquatica* and *D. zeae*. All the bacteriophages from orchid tissue showed identical host-range profiles. They were active against *Dickeya* sp. UDL-3 isolates, *Dickeya* sp. MK7 and *D. dadantii* NCPPB 898. However, the bacteriophages isolated from wastewater showed variability in their host specificities. The host ranges of the bacteriophages BF-CIM5/14, BF-CIM6/14, BF-CIM8/14, and BF-CIM10/15 were very similar, and their specificities were close for both *Dickeya* spp. UDL-3 and UDL-4. However, substantial differences in specificities against *Dickeya* spp. from the bacteria collection here were observed. The bacteriophages BF-CIM8/14 and BF-CIM10/14 did not only infect *D. aquatica* and *D. zeae*. Conversely, infection by the bacteriophages BF-CIM5/14 and BF-CIM6/14 was limited to *Dickeya* sp. MK7 and weakly to *D. solani* GBBC 500 and *D. solani* GBBC 2040. Finally, all three bacteriophage isolates from the BF-CIM1/14 group showed a uniform infection profile; they were active only against *Dickeya* sp. UDL-4 isolates *D. solani* GBBC 500 and *D. dianthicola* LMG 2458 (**Table 4**).

**Table 4:** Host range of bacteriophages isolated against UDL-3 and UDL-4 isolates.

Bacterial species and strain	Origin		Bacteriophage cone of lysis			
	Source country	Year	Waste water^a^			Orchid^b^
			BF-CIM1/14	BF-CIM5/14, BF-CIM6/14	BF-CIM8/14, BF-CIM10/14	BF25/12
***Dickeya* spp. UDL-3**						


B16	Slovenia	2010	No	Clear	Clear	Clear


COB 2/12	Slovenia	2012	No	Clear	Clear	Clear


COB 7/12	Slovenia	2012	No	Clear	Clear	Clear


COB 8/12	Slovenia	2012	No	Clear	Clear	Clear


COB 10/12	Slovenia	2012	No	Clear	Clear	Clear


COB 11/12	Slovenia	2012	No	Clear	Clear	Clear


COB 13/12	Slovenia	2012	No	Clear	Clear	Clear


COB 15/12	Slovenia	2012	No	Clear	Clear	Clear


COB 16/12	Slovenia	2012	No	No	Clear	Clear


COB 17/12	Slovenia	2012	No	Clear	Clear	Clear


***Dickeya* spp. UDL-4**						


S1	Slovenia	2012	Clear	Clear	Clear	No


COB9/12	Slovenia	2012	Clear	Clear	Clear	No


COB12/12	Slovenia	2012	Clear	Clear	Clear	No


COB14/12	Slovenia	2013	Clear	Clear	Clear	No


***Dickeya* spp. from bacteria collection**						


*D. solani* NIB Z 1821 (GBBC 500)	NA	NA	Clear	Weak	Clear	No


*D. solani* NIB Z 1823 (GBBC 2040)	Belgium	2007	No	Weak	Clear	No


*D. dianthicola* NIB Z 1824 (LMG 2485)	United Kingdom	1956	Clear	No	Clear	No


*D. dieffenbachiae* NIB Z 1826 (LMG 25992)	United States	1957	No	No	Clear	No


*D. zeae* NIB Z 1827 (LMG 2497)	United States	1966	No	No	No	No


*D. dadantii* NIB Z 2131 (NCPPB 898)	Comoros Is.	1961	No	No	Clear	Clear


*Dickeya* spp. NIB Z 2132 (NCPPB 3274)	St. Lucia	1983	No	No	Clear	No


*D. aquatic* NIB Z 2133 (NCPPB 4580)	United Kingdom	2008	No	No	No	No


*Dickeya* spp. MK7 NIB Z 2211	Scotland	NA	No	Clear	Clear	Clear


**Other bacteria isolated from infected orchid tissue**						


COB3/12	Slovenia	2012	No	No	No	No


COB4/12	Slovenia	2012	No	No	No	No


COB 5/12	Slovenia	2012	No	No	No	No


COB 6/12	Slovenia	2012	No	No	No	No


**Bacteria isolates from asymptomatic orchids**						


COB 1/13	Slovenia	2013	No	No	No	No


COB 2/13	Slovenia	2013	No	No	No	No


COB 3/13	Slovenia	2013	No	No	No	No


COB 4/13	Slovenia	2013	No	No	No	No


COB 5/13	Slovenia	2013	No	No	No	No


COB 6/13	Slovenia	2013	No	No	No	No


COB 7/13	Slovenia	2013	No	No	No	No


COB 8/13	Slovenia	2013	No	No	No	No


COB 9/13	Slovenia	2013	No	No	No	No


COB 10/13	Slovenia	2013	No	No	No	No


***Pectobacterium cypripedii***						


NIB Z 1559 (NCPPB 750)	United States	1950	No	No	No	No


NIB Z 1560 (NCPPB 751)	United States	1950	No	No	No	No


NIB Z 1562 (NCPPB 3129)	United States	NA	No	No	No	No


NIB Z 1563 (NCPPB 3889)	United Kingdom	1994	No	No	No	No


NIB Z 1564 (NCPPB 4059)	United Kingdom	1999	No	No	No	No


NIB Z 1529 (NCPPB 3004)	United States	NA	No	No	No	No
**Enterobacteriaceae from culture collection**						
*Pantoea agglomerans* NIB Z 25	Slovenia	2002	No	No	No	No
*Proteus vulgaris* NIB Z 856	NA	NA	No	No	No	No
*Proteus mirabilis* NIB Z 857	NA	NA	No	No	No	No
*Delftia acidovorans* NIB Z 858	NA	NA	No	No	No	No
*Erwinia billingiae* NIB Z 1142 (DSM 17872)	United Kingdom	1999	No	No	No	No
*Erwinia tasmaniensis* NIB Z 1143 (DSM 17950)	Australia	2006	No	No	No	No
*Enterobacter* sp. NIB Z 1701 (NCCPB 4168)	NA	NA	No	No	No	No
*E. coli* NIB Z 2036	NA	NA	No	No	No	No
*E. coli* NIB Z 2037 (DSM 423)	NA	NA	No	No	No	No
*E. coli* NIB Z 2038 (DSM 13127)	NA	NA	No	No	No	No
*E. coli* NIB Z 2039 (APEC_FUW1)	NA	NA	No	No	No	No
*E. coli* NIB Z 2040 (DSMZ 613)	NA	NA	No	No	No	No

^a^*Bacteriophages isolated from wastewater; ^b^bacteriophage isolated from diseased orchids; all other bacteriophages isolated from plant material were characteristically the same to the characterized bacteriophage BF25/12. The host range for one of the representative bacteriophages from each isolated group is given*.

Based on the plaque morphology, RFLP profiles, host ranges and transmission electron microscopy analysis, three different bacteriophages were isolated. Two were derived from the wastewater and were members of the *Myoviridae* and *Siphoviridae* families. The third bacteriophage belonged to the *Podoviridae* family and was obtained from the soft rot orchid tissue. Based on the characteristics of these bacteriophages, bacteriophage BF25/12 was chosen for further analysis.

### Transmission Electron Microscopy of Bacteria–Bacteriophage Interactions

**FIGURE 3:**
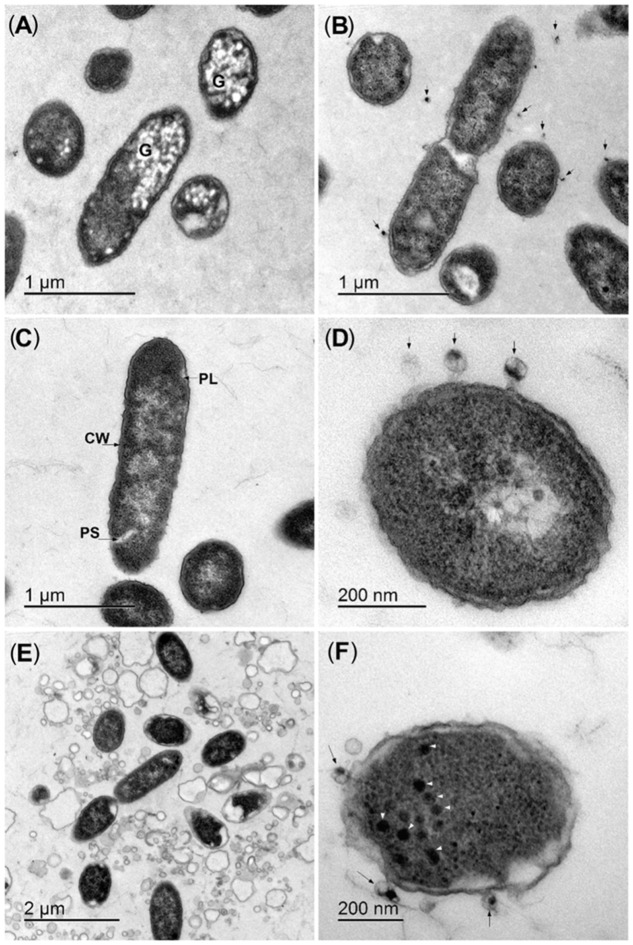
Representative ultrastructure analyses of bacteria–bacteriophage interactions. **(A,B)** Strain UDL-4 before **(A)** and after **(B)** mixing with bacteriophage BF25/12 (arrows). **(C,D)** Strain UDL-3 before **(C)** and after **(D)** mixing with bacteriophage BF25/12 (arrows). **(E,F)** Lysed UDL-3 bacteria from the border of a plaque **(E)** and new bacteriophages in the cytoplasm (arrowheads) **(F)**. G, intracellular granules; CW, cell wall; PL, plasmalemma; PS, periplasmic space.

The interactions of the selected bacteriophage BF25/12 with the host bacteria were examined at the ultrastructure level. The interactions of BF25/12 and the UDL-4 bacteria activated granule degradation in the bacteria cells without bacterial lysis. Most of these UDL-4 bacteria contained electron-lucent intracellular granules (20–70 nm in diameter), which were similar to intracellular inclusion bodies (**Figure 3A**; Alič et al., 2017). When bacteria UDL-4 and bacteriophages BF25/12 were incubated together for 15 min, these granules almost completely disappeared, and there were bacteriophages attached to the bacterial surface (**Figure 3B**). Mixtures of *Dickeya* sp. UDL-3 (**Figure 3C**) and BF25/12 also showed the attachment of the bacteriophages to the bacterial surface (**Figure 3D**). Based on the plaque assays, the borders of the plaques were analyzed. Most of the UDL-3 cells here were lysed, and the plasmalemmas of the resting cells were detached (**Figure 3E**). New bacteriophages could also be seen in the cytoplasm of some of the bacteria (**Figure 3F**).

### Genome of Bacteriophage BF25/12

**FIGURE 4:**
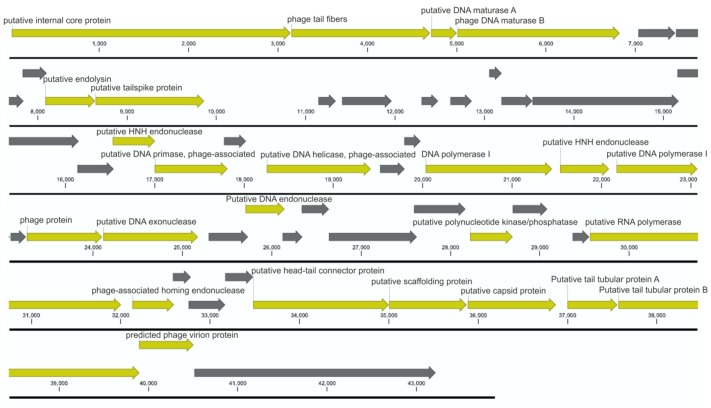
Annotated BF25/12 bacteriophage genome. According to the annotation, the genome does not contain any genes encoding antibiotic resistance or toxins. The open reading frames coding for structural proteins, proteins involved in bacteriophage replication, and other conserved proteins are shown in yellow, and those coding for hypothetical proteins are shown in gray. Unit of the presented genome annotation is in bp.

The genome of bacteriophage BF25/12 was additionally characterized using genome sequencing and automatic annotation. BF25/12 contains linear double-stranded (ds)DNA that is 43,872 bp long with 52.2% GC, which supports its classification into the *Podoviridae* family. The genome of bacteriophage BF25/12 contains 52 open reading frames that were determined with RAST (Aziz et al., 2008; Overbeek et al., 2014; Brettin et al., 2015). They mainly coded for hypothetical proteins (27 genes), along with structural proteins and proteins involved in bacteriophage replication. Furthermore, a single putative endolysin gene was identified, corresponding to the halo effect observed in plaque morphology. However, no genes that encode antibiotic resistance or toxins were detected (**Figure 4**). The annotated BF25/12 genome sequence is available in GenBank under Accession No. KT240186.

The genomic sequence of bacteriophage BF25/12 was compared to characterized genera from the *Podoviridae* family using the PASC tool (Bao et al., 2014). BF25/12 showed low identity (<3%) to any member of the *Picovirinae* or *Sepvirinae* subfamilies. However, it shared higher identity to the *Autographivirinae* subfamily, especially *Klebsiella* virus F19 from the Kp34 virus genus (33% identity). Based on the pairwise identity analysis conducted, the BF25/12 genome was 57–62% identical to three unassigned *Pectobacterium* bacteriophages, *Pectobacterium* phages PP16, PP90, and Peat1. The results correspond to the nBLAST analysis. The assembled BF25/12 genome showed substantial similarity (74–79% identity and 21–56% query coverage) to five bacteriophage genomes in the BLAST database. All five genomes belonged to *Pectobacterium* bacteriophages, four of which were assigned to the *Podoviridae* family, such as BF25/12, and the last was unclassified. The closest hit represented *Pectobacterium* bacteriophage PP16 (79% identity over 56% query coverage).

### UV Radiation Stability

Bacteriophage BF25/12 was not able to survive a 2-min exposure to UV-C light. The reduction of active bacteriophages was 10^5^, corresponding to almost 100% in the first minute of exposure.

### pH Stability

Bacteriophage pH stability analysis in SMG buffer and sterile demineralised water at two different bacteriophage concentrations (10^5^ and 10^3^ pfu/mL) showed that bacteriophage BF25/12 pH stability is affected by both its concentration and environment (**Figure 6**). Bacteriophage BF25/12 lost its infectivity completely at pH 3, regardless of the incubation media (SMG buffer or sterile demineralised water). It showed maximum stability at pH 7 and 9, which indicated the favorable pH for bacteriophage BF25/12 is neutral to mildly alkaline. Furthermore, no significant difference in the decrease of bacteriophage titer could be detected between the tested incubation media or bacteriophage concentrations after 24 h incubation at pH 7 and 9. At higher concentrations in SMG buffer, a mildly acidic pH (pH 5) did not cause significant phage de-activation. Bacteriophage incubated in SMG buffer completely lost its infectivity at pH 11 after 24 h incubation, but its infectivity was not affected in sterile demineralised water in the same conditions. The exposure of highly concentrated bacteriophage lysate (10^8^ pfu/mL) to acidic (pH 3) and alkaline (pH 11) environments for 24 h had almost no detectable effect on bacteriophage survival and infectivity (data not shown).

### Temperature Stability

**FIGURE 5:**
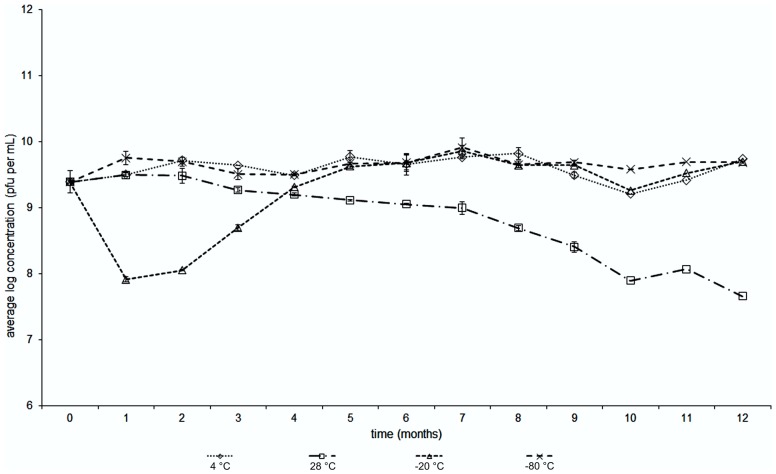
Temperature stability with time of *Dickeya* spp. bacteriophage BF25/12 incubated at temperatures of +4, +28, –20 and –80°C (as indicated).

The stability of the bacteriophage BF25/12 active against *Dickeya* spp. was monitored at different temperatures over a year. It proved to be stable at 4°C, as the changes in the bacteriophage titers were negligible, which indicated this is a favorable temperature for long-term bacteriophage storage (**Figure 5**). The bacteriophage stability at -80°C appeared similar to that at 4°C. However, temperatures of -20°C proved to be inappropriate for long-term storage due to the notable decline of the bacteriophage titer. This unusual decrease in the bacteriophage titer at -20°C was seen after the first 3 months of storage. In the following months, the bacteriophage concentration returned to the initial bacteriophage titer. The least favorable temperature tested was 28°C.

**FIGURE 6:**
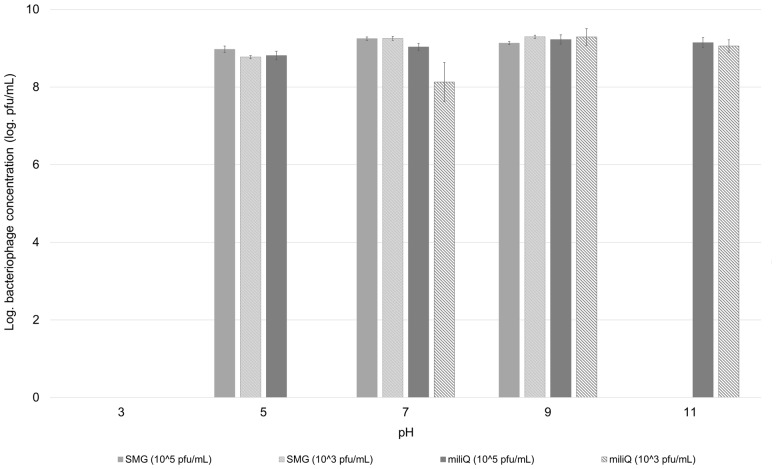
Effect of pH on stability of bacteriophage BF25/12. Stability was tested in SMG buffer and sterile demineralised water at two different bacteriophage concentrations, 10^5^ and 10^3^ pfu/mL. Concentration of bacteriophages presented in the graph was calculated to the level of bacteriophage stock, used for preparation of testing dilutions.

### Bacterial Resistance

**FIGURE 7:**
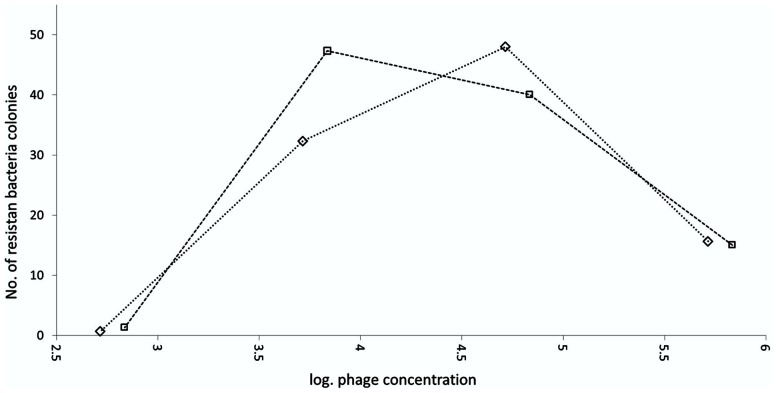
Emergence of *Dickeya* spp. B16 resistant to bacteriophage BF 25/12. Experiment was performed as two technical repeats, shown on graph as squares and diamonds.

The emergence of resistance against bacteriophage BF25/12 was determined for host bacteria *Dickeya* sp. B16. The number of growth-resistant bacterial colonies depended greatly on the bacteriophage concentration in the medium (**Figure 7**). The highest rate of bacterial resistance development against bacteriophage BF25/12 was at the final bacteriophage concentration of between log 4 and log 5 and the initial bacteria concentration of log 8. Bacteriophage concentrations <log 4 and >log 5 provided lower selection pressure, which resulted in the decreased resistance of the bacterial colonies.

## Discussion

In the present study, we isolated bacteriophages active against the plant pathogens of *Dickeya* spp. that cause the soft rot of orchids, which is a destructive disease for *Phalaenopsis* production sites. Bacteriophages were isolated from two different sources, diseased *Phalaenopsis* tissue and wastewater. For the latter, CIM monolith chromatography was used for bacteriophage particle concentration. CIM monolith chromatography has been reported as an effective technique for virus concentration and for purification of virus particles (Steyer et al., 2015). However, we believe this is the first report that describes the use of CIM monolith chromatography as a technique for the isolation of infectious bacteriophages from environmental samples. It proved to be very efficient for the concentration of bacteriophages from environmental samples, which allowed more efficient bacteriophage enrichment on the host bacterium and therefore better yields in the bacteriophage isolation. Seventy-two percent of bacteriophages active against *Dickeya* spp. obtained in this study were isolated from wastewater using CIM monolith chromatography, indicating the presence of viable *Dickeya* spp. bacteria in wastewater or in plants that are in direct contact with wastewater. Furthermore, different sources of bacteriophage isolation resulted in different bacteriophages from different families and with different RFLP patterns, although they showed similar host-specificity profiles. The isolation of the bacteriophages from orchid samples was carried out in summer (i.e., July and September) when the highest concentrations of the bacteria are expected. The isolates from wastewater were obtained from late spring to early autumn, excluding July and September. The attempts to isolate bacteriophages from soil were not successful, regardless of the month when the sampling was carried out (data not shown). Therefore, these differences cannot be explained in terms of seasonal changes, which indicates the specificity of each of these bacteriophage sources.

However, the bacteriophages active against *Dickeya* spp. that showed high specificities can be used as biocontrol agents, as the treatment of a diseased orchid with these bacteriophages can specifically eliminate the pathogenic bacteria while leaving the normal microbiota unharmed. Adriaenssens et al. (2012) and Czajkowski et al. (2014, 2015) reported the isolation of bacteriophages specifically against the aggressive potato pathogen *D. solani*, bacteriophages with multiple hosts across *Dickeya* spp. and broad host-range bacteriophages against *Pectobacterium* spp. and *Dickeya* spp. All the bacteriophages active against *Dickeya* spp. described in the literature belong to the order *Caudovirales* as members of the *Myoviridae* family (Adriaenssens et al., 2012; Czajkowski et al., 2014, 2015; Soleimani-Delfan et al., 2015), and the first report about a bacteriophage from the *Siphoviridae* family was made recently (Soleimani-Delfan et al., 2015). However, the present study represents the first report of bacteriophages active against *Dickeya* spp. from the *Podoviridae* family. The bacteriophages from different families show some small differences in their specificities. All the bacteriophages from the *Podoviridae* (BF21/12 – BF25/12) and *Myoviridae* (BF-CIM5/14 – BF-CIM10/14) families infected all *Dickeya* isolates in the present study, as did the aggressive potato pathogen *D. solani* and other reference *Dickeya* strains, excluding *D. paradisiaca* and *D. aquatica*. However, as members of the *Siphoviridae* family, bacteriophages BF-CIM1/14 showed different host ranges. The activity against the *Dickeya* sp. UDL-3 isolates varied and was relatively low, although all the *Dickeya* sp. UDL-4 and *D. solani* strains were highly susceptible, which supports a relationship between those two *Dickeya* spp. (Alič et al., 2017).

The selected bacteriophage here, BF25/12, was characterized at the genome level. It showed no similarities to any previously described bacteriophages that are active against *Dickeya* spp. However, homologous bacteriophages deposited in GenBank were identified using BLAST and PASC analysis. The BF25/12 genome showed the highest similarity to bacteriophages active against *Pectobacterium* species characterized as members of the *Podoviridae* family, such as BF25/12. *Pectobacterium* bacteriophages did not share similarity to BF25/12 across the genome; rather, the homology was limited to sections of the genome. This lack of genetically similar bacteriophages can be explained on the basis that this is the first member of the *Podoviridae* family among bacteriophages active against *Dickeya* spp. Bacteriophage BF25/12 did not show significant similarities to any currently described and characterized *Podoviridae* genus at the genome level. Based on the genomic and morphologic characteristics, it is closest to genera in the *Autographivirinae* subfamily, which includes the T7 virus genus and the *Escherichia coli* model bacteriophage T7. The bacteriophage T7 virion consists of a head with a diameter of 55 nm and a 23-nm long tail (Steven and Trus, 1986). The size of the T7 virus particle corresponds to the measurements of the BF25/12 virion (head approximately 56 nm × 52 nm and tail 16 nm × 12 nm). The BF25/12 genome size of 43.8 kbp and the 52.2% GC content matches the small T7 bacteriophage genome (around 40 kbp and 48.4% GC content; Dunn et al., 1983). Furthermore, T7 and BF25/12 have an almost identical number of coding sequences in the genome—50 and 52 genes, respectively (Dunn et al., 1983). A genome analysis showed the suitability of BF25/12 for field-application studies due to its lack of genes coding for the bacteriophage lysogenic cycle and of genes encoding toxins or antibiotic resistance.

Bacteriophage BF25/12 showed differences in activity against the UDL-3 and UDL-4 bacteria, which are genetically very similar bacterial isolates. Differences in the infection of these two isolates were observed under transmission electron microscopy, which resulted in some phenomena that have not been reported previously. The interactions of the bacteriophage with the UDL-4 bacteria activated granule degradation in the bacterial cells, which are similar to intracellular storage granules, with no bacterial lysis. The structure and function of these observed inclusions remain unknown, although a type of intracellular metabolic reserve is most likely. Some metabolic inclusions that function as a metabolic reserve are directly connected to the persistence of bacteria. Glycogen metabolism has been linked to bacterial survival in the environment, as well as to colonization and virulence (reviewed in Wilson et al., 2010). Moreover, in *Pseudomonas oleovorans*, it was shown that the metabolism of intracellular polyhydroxyalkanoates granules contributes to bacterial cell survival. The wild-type strain *P. oleovorans*, which is able to degrade intracellular polyhydroxyalkanoates, showed enhanced resistance to stress agents compared to the corresponding mutant strain incapable of the depolymerisation of polyhydroxyalkanoates (Ruiz et al., 2001). This observed granule degradation in UDL-4 cells might represent a bacteria stress response to the bacteriophage infection process, whereby the metabolic inclusions might enhance the environmental survival of the bacteria and therefore limit the development of the bacteriophage infection. However, a correlation between bacterial resistance to bacteriophage lysis and intercellular storage granule degradation has never been described.

Some of the main challenges in bacteriophage applications are environmental factors, including temperature, pH and UV radiation. Temperature can affect the ability of bacteriophages to lyse bacteria and their survival on the plant surface (Jones et al., 2007). Monitoring bacteriophage stability here revealed decreased bacteriophage titers at 28°C. The observed outcome corresponds to a study by Jepson and March (2004), who reported a decrease in liquid bacteriophage λ stock at 37°C over 120 days to under the limit of detection. However, the decline in the viability of bacteriophage BF25/12 was only 27.34% in 1 year, which indicates its applicability due to this appreciable stability at the implementation temperature. As expected, the bacteriophage titer at 4°C remained stable over the observed period. Bacteriophage BF25/12 belongs to the tailed bacteriophages that are known to be extremely viable at 4°C, and some can retain viability for up to 10–12 years (Jepson and March, 2004; Jończyk et al., 2011). Storing bacteriophages at -20°C is not recommended, as the formation of ice crystals can damage or destroy bacteriophage particles (Jończyk et al., 2011). Furthermore, suboptimal pH can inactivate bacteriophages; therefore, the stability and pH optimum of bacteriophages is very important in plant disease control applications (Jones et al., 2007). Bacteriophage BF25/12 was sensitive to acid pH and showed maximum stability in neutral and alkaline conditions. Only bacteriophages incubated in sterile demineralised water were stable at pH 11, indicating the influence of the incubation medium to bacteriophage stability at certain pH. It has been reported that buffer and salt concentration can affect bacteriophage activity (Chow et al., 1971; Burnet and Stanley, 2013). Chow et al. (1971) reported higher stability of bacteriophage Xp12 in Tris buffer at a higher pH; the effect is more prominent at higher ionic strengths. However, the effect was studied only for the pH range from 7 to 9. The most damaging environmental factor for bacteriophage stability is UV radiation (Jones et al., 2007), and, as reported for other phages, BF25/12 is highly susceptible to UV radiation (Jones et al., 2007).

The occurrence of resistant bacteria mutants represents a major concern when using bacteriophages as biocontrol agents (reviewed in Jones et al., 2007). Here, the dependence between bacteriophage concentration and the development of bacterial resistance was optimum from log 4 to log 5 pfu/mL. These data indicate the importance of using suitable bacteriophage concentrations for bacteriophage treatments and the danger of using a single bacteriophage in any active treatment approach. The active bacteriophage used in this treatment undergoes extensive reproduction above the threshold needed to kill the bacteria through secondary infection (Payne et al., 2000). Extensive enrichment of bacteriophage-resistant bacteria might occur if the initial bacteriophage concentration used in any active approach is below the observed optimum for resistance development; for *Dickeya* spp. B16 and BF25/12, this was below log 4. However, the emergence of resistant bacteria during bacteriophage biocontrol can be reduced by the use of bacteriophage cocktails instead of individual bacteriophages (reviewed in Jones et al., 2007).

In terms of the applicability of the bacteriophages defined in the present study, it will be necessary to determine the infection parameters (e.g., adsorption rate, one-step growth assay, burst size) to determine the potential effectiveness of this bacteriophage as a biocontrol. The combination of these isolated bacteriophages could in future form an intriguing bacteriophage cocktail due to the combination of the three different bacteriophage families with similar specificities. However, the biocontrol properties of the bacteriophages should be evaluated by pot or field experiment. The dynamic approach of the bacteriophage biocontrol agents requires extensive knowledge of a specific bacteria–bacteriophage system. However, bacteriophages can also be used in combined biocontrol management strategies, as they enable a temporary decrease in bacterial inoculum and therefore the greater efficiency of other substances used in bacterial disease control and treatment. Presently, isolated phages represent an important research tool that can provide adequate knowledge of bacteria–bacteriophage systems and dynamics, which is essential for the applicability of bacteriophages.

## Author Contributions

Conception and design of the experiments: ŠA, TN, TD, and MR. Performing experiments, analysis and interpretation of the data: ŠA, TN, MT-Ž, and TD. Provided CIM virus concentrates: NR. Drafting the work and writing the paper: ŠA, TN, MT-Ž, and TD. Revising the work critically for important intellectual content: ŠA, TN, MT-Ž, MP, MR, and TD. All authors read and approved the final manuscript.

## Conflict of Interest Statement

The authors declare that the research was conducted in the absence of any commercial or financial relationships that could be construed as a potential conflict of interest.
